# Technical Note: Use of automation to eliminate shift errors

**DOI:** 10.1002/acm2.12830

**Published:** 2020-02-10

**Authors:** Elizabeth L. Covington, Richard A. Popple, Rex A. Cardan

**Affiliations:** ^1^ Department of Radiation Oncology University of Alabama – Birmingham Birmingham AL USA

**Keywords:** automation, RO‐ILS, scripting, shift errors

## Abstract

**Purpose:**

To create automated tools within the treatment planning system (TPS) that eliminate the common error pathway of providing incorrect shift instructions to therapists.

**Materials/Methods:**

Two scripts were created within the TPS using the Eclipse API (Varian Medical Systems, Palo Alto, CA). One script detects whether or not the user origin has been placed correctly at the intersection of the simulation markers while the other calculates a shift instruction sheet that can be printed for treatment.

**Results:**

Analysis of our RO‐ILS database identified eight errors caused by improper setting of the user origin in the treatment planning system. The user origin script flagged all of the treatment plans for markers inconsistent with user origin. Automated calculation of shifts eliminated the error pathway of miscalculating or transcribing shift values.

**Conclusion:**

Automation can eliminate the common error pathway of providing the wrong shifts to therapists. The scripts have been made available as open‐source software for implementation at other radiotherapy clinics.

## 1 INTRODUCTION

Incident learning systems are a key component of safety culture in the medical field.^1^, ^2^, ^3^, ^4^, ^5^ The Radiation Oncology Incident Learning System (RO‐ILS) is an incident learning system sponsored by both ASTRO and AAPM which enables users to submit radiation oncology‐related incidents to a national database.^6^, ^7^ With this database, RO‐ILS is able to review large numbers of events and provide education to clinics on the types of incidents reported via quarterly reports and case studies. RO‐ILS can also serve as an institution’s primary ILS for review and management of incidents.

A recent paper by Ezzell et al. looked at the highest priority reports from RO‐ILS.^6^ Out of over two thousand reports, 396 were deemed the highest priority and coded with up to three keywords. When analyzing this subset of high priority events, 44% were found to belong to the following error pathways: “problematic plan approved for treatment,” “wrong shift instructions given to therapists,” and “wrong shift performed at treatment.” During an analysis of our own RO‐ILS data, we found that one of our highest occurring errors was also “wrong shift instruction given to therapists.”

In this paper, we present two tools to eliminate the pathway of “wrong shift instructions given to therapists” using application programming interface (API) scripts within the treatment planning system. The first tool is an automated check that determines whether the user origin has been set correctly as specified by the location of the CT simulation markers. The second tool automatically calculates the shifts which can be printed or saved to portable document format (PDF) for therapists to use for the first treatment. These solutions are available in an open‐source library on GitHub (https://github.com/), an online repository that allows the sharing of software, for readers to implement.

## 2 METHODS

**Figure 1:**
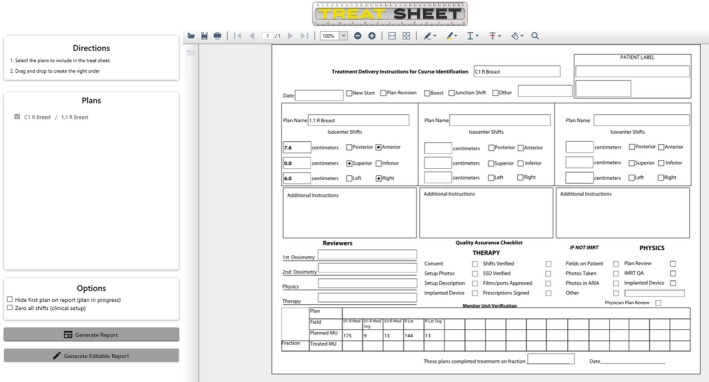
User interface of the “Treat Sheet” script with example treatment plan loaded. All available plans are listed on the left with the ability to calculate shifts between plans.

Our simulation workflow uses three radiopaque markers to indicate the location of the simulation marks on the CT scan. Once the CT scan is imported into the TPS, the dosimetrist places the user origin of the treatment plan at the intersection of these three markers. If the isocenter is moved from the user origin, shifts in the lateral, anterior/posterior, and superior/inferior direction are provided to therapists. To calculate these shifts, a script was developed using the Eclipse application programming interface (API) (Varian Medical System, Palo Alto, CA). This script finds the difference in the user origin and isocenter locations and performs a DICOM to IEC transformation to calculate the shifts needed for treatment. The program takes patient orientation (e.g. head first supine) into consideration when calculating the shifts and can also calculate shifts between treatment plans created on the same CT scan. This information can be printed or saved to PDF for the therapists to use during the first treatment. These shifts are included with a pre‐treatment QA checklist for therapy, and the document is known as the "Treat Sheet." An example of Treat Sheet is shown in Fig. 1.

**Figure 2:**
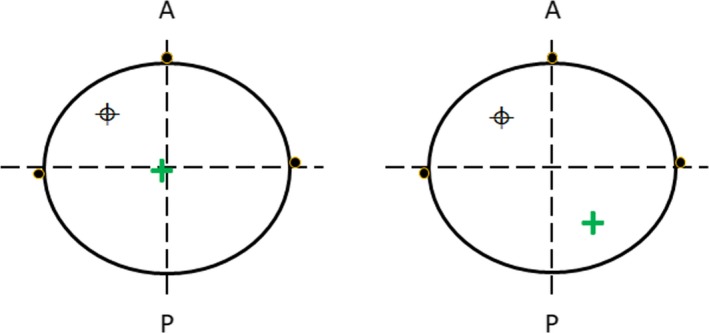
(Left) User origin has been set within tolerance of the intersection of CT markers and will pass an automatic check that confirms the intersection of marker contours and user origin. Average anterior (A) and posterior (P) coordinate will be used if contours aren’t aligned. (Right) User origin not set correctly and resulting shifts to isocenter () will be incorrect and will be flagged by script.

The “Treat Sheet” script relies on the user origin. If the planner does not correctly set the user origin at the intersection of the markers, the shift instructions will be incorrect. Therefore, a second tool was developed to ensure that the user origin has been placed at the CT simulation marks. This is done by having the CT markers contoured with contour names following a specified naming convention. The script finds the average y (anterior/posterior) and z coordinate (superior/inferior) for the two lateral markers. The x coordinate (lateral) is defined by the marker placed on either the anterior or posterior surface of the patient. The intersection of these coordinates is used to define the user origin and verified again the location within the TPS as shown in Fig. 2. The check will flag the user if the script determined user origin differs from the treatment plan user origin by a set tolerance. This check is part of a larger suite of plan quality checks known as XCheck.

A retrospective analysis of our RO‐ILS incidents was performed to identify all errors that included shift errors. Incidents were stratified as either being caused by incorrect user origin or other causes. For those caused by incorrectly set user origin, XCheck was run to determine if the script would have flagged the user origin, therefore preventing the error.

## 3 RESULTS

**Figure 3:**
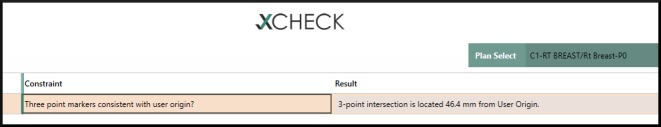
Example of script that flags users that the origin has not been set at the intersection of the markets set at simulation.

Out of 68 events reported into our RO‐ILS system, eight were identified as shift errors. Out of the eight reported, seven were found to be caused by an incorrectly set user origin. This was tied for our highest error pathway along with prescription errors. The eighth shift error was caused by an accidental override of the shift values by the dosimetrist in the Treat Sheet script. Overriding of computed values was part of the program design to allow for clinical in‐room setups (e.g. electron treatments). Xcheck was run on six of the seven treatment plans known to have the user origin error. The software correctly flagged all as having user origin not set at the intersection of the markers. See Fig. 3 for an example of the user interface that alerts staff of the error. Offsets ranged from 2.7 to 82.3 mm. Note that one treatment plan could not be tested due to the medical record number not being recorded into RO‐ILS.

These two scripting tools presented have been made available online for other institutions to implement. The script used to calculate the transformation between DICOM and IEC coordinates is available on GitHub (https://github.com/rexcardan) in a larger suite of Eclipse scripting tools, called XCheck. The origin checker has also been released in order for clinics to have access to the full solution for preventing shift errors. These tools are part of a growing suite of open source software solutions used to improve safety and standardization in radiotherapy clinics developed at our institution.^8^

## 4 DISCUSSION

The Treat Sheet is an independent script that is run per patient by dosimetry once the plan has been approved by the physician. The generated output document is printed and provided to the therapists and serves as their pre‐treatment checklist and shift sheet. The user origin check is one of many plan quality checks in XCheck, which is run throughout the treatment planning process. The script is initiated by the user in the TPS and takes approximately 60‐90 seconds to run, depending on the number of checks selected. Dosimetrists are encouraged to run XCheck before planning to catch errors early, then again before physician approval to prevent replanning. Physicists also run Xcheck on each patient during the physics pre‐treatment plan review.

As discussed in numerous publications, processes that require manual calculation or transcription are prone to error.^1^, ^6^, ^9^ The fault tree for providing wrong shifts to therapists by Ezzell et al. shows three major pathways including “error in manually calculating or transcribing” shifts. Common errors were reversing directions and wrong units (e.g. centimeters instead of millimeters). The creation of the Treat Sheet script can eliminate this error, since no manual calculation or transcribing of shifts is needed due to the automatic calculation and printing of shifts. Shifts are hardcoded to report in centimeters to avoid incorrect units. Reviewing the previous three years of RO‐ILS incidents found that only one shift error in RO‐ILS was due to this pathway. This was due to a dosimetrist erroneously overriding the computed shift.

While the calculation and transcription pathway was nearly eliminated, incidents continued to occur due to another pathway in this fault tree: “Origin on planning CT does not match set‐up point.” Since the user origin is manually set at the intersection of the CT markers by the dosimetrist, it is prone to failure. Out of the eight shift errors report, seven were due to this error pathway which was the impetus for creating the second script to verify user origin. While it does not eliminate the occurrence of the error, the check will flag the dosimetrist and physicist thereby increasing the detectability of the error and providing the opportunity for correction. The third pathway mentioned, “Wrong IGRT reference,” was not the root cause of any of our reported incidents.

## 5 CONCLUSIONS

Two scripts have been developed to eliminate the error pathway of “wrong shift instructions given to therapists.” The Treat Sheet has eliminated errors due to manual calculation of shifts and/or transcription errors. The second script, which detects whether the treatment planning system user origin has been correctly set at the intersection of the simulation markers, automatically detects errors from another error pathway and flags the user. With these scripts, users can eliminate or greatly reduce errors from one of the highest priority errors reported into the RO‐ILS database.

## CONFLICTS OF INTEREST

No conflicts of interest.
